# Crop Species Mechanisms and Ecosystem Services for Sustainable Forage Cropping Systems in Salt-Affected Arid Regions

**DOI:** 10.3389/fpls.2022.899926

**Published:** 2022-05-24

**Authors:** Dennis S. Ashilenje, Erick Amombo, Abdelaziz Hirich, Lamfeddal Kouisni, Krishna P. Devkota, Ayoub El Mouttaqi, Abdelaziz Nilahyane

**Affiliations:** African Sustainable Agriculture Research Institute (ASARI), Mohammed VI Polytechnic University (UM6P), Laayoune, Morocco

**Keywords:** species salinity tolerance, photosynthesis, relative yield, synergies, forage cropping systems

## Abstract

Soil salinity limits crop productivity in arid regions and it can be alleviated by crop synergies. A multivariate analysis of published data (*n* = 78) from arid and semiarid habitats across continents was conducted to determine the crop species mechanisms of salinity tolerance and synergies relevant for designing adapted forage cropping systems. Halophyte [*Cynodon plectostachus* (K. Schum.) Pilg.] and non-halophyte grasses (*Lolium perenne* L. and *Panicum maximum* Jacq.) clustered along increasing soil salinity. Halophytic grasses [*Panicum antidotale* Retz. and *Dicanthum annulatum* (Forssk.) Stapf] congregated with *Medicago sativa* L., a non-halophytic legume along a gradient of increasing photosynthesis. Halophytic grasses [*Sporobolus spicatus* (Vahl) Kunth, and *Cynodon plectostachyus* (K. Schum.) Pilg.] had strong yield-salinity correlations. *Medicago sativa* L. and *Leptochloa fusca* L. Kunth were ubiquitous in their forage biomass production along a continuum of medium to high salinity. Forage crude protein was strongly correlated with increasing salinity in halophytic grasses and non-halophytic legumes. Halophytes were identified with mechanisms to neutralize the soil sodium accumulation and forage productivity along an increasing salinity. Overall, halophytes-non-halophytes, grass-forbs, annual-perennials, and plant-bacteria-fungi synergies were identified which can potentially form cropping systems that can ameliorate saline soils and sustain forage productivity in salt-affected arid regions.

## Introduction

Forage crops and rangeland species have immense potential to rehabilitate and improve the natural ecosystem services besides providing forage supply to livestock and wildlife. They maintain soil health by regulating environments against the effect of climate change and pollution. In arid regions, these benefits from vegetation have been jeopardized by adverse environmental conditions, particularly endemically marginal precipitation (Antipolis, 2003). Traditional interventions such as selection and cultivation of drought-tolerant species and irrigation strategies have helped to augment forage production in these areas (Qadir et al., 2007). However, these measures have implications in aggravating salt accumulation in soils and diminishing ecosystem services associated with forage crops.

Soil salinity is a major problem limiting forage productivity in a wide range of agroecosystems. An estimated 1,125 million ha of land distributed across different continents is degraded by salinity (Hossain, 2019). The problem of salinity is unique in different ecosystems with greater prevalence in arid and semi-arid zones (Antipolis, 2003). Salt accumulation from seawater intrusion is endemic to coastal areas. However, mismanagement of agronomic practices particularly over-irrigation using brackish water triggers capillary and evapotranspiration and as a result, unfavorable amounts of salts accumulate in the soil profile. Saline soils are characterized by electrical conductivity (EC) exceeding 4 dS m^–1^ and sodium accumulation greater than 15% (Allison et al., 1954). Salinity can occur simultaneously with alkalinity, where accumulation and hydrolysis of Na and NaCO_3_ release OH^–^ ions which elevate pH above 8.5 and consequently phosphorus, calcium, magnesium, and zinc precipitate and are less available to plants (Choudhari and Kharche, 2018; Wahba et al., 2019). High pH (>9) and Na^+^ ions relatively thicken the saline layer and mediate the enhanced repulsive force in soil intermicellar layer which causes soil particle dispersion (Zhou and Yu, 2016). Dispersion disaggregates soil layers and predisposes organic carbon to mineralization (Wong et al., 2010), enhances compaction, obstructs soil water infiltration and as consequence soils are easily swept off by runoff (Shrivastava and Kumar, 2015). The excess NaCl concentration in soil solution inhibits microbial enzymes and therefore limits C and N cycling (Rietz and Haynes, 2003), net primary production, and turnover of crop residues (Yannarell and Pearl, 2007). At the ecological scale, soil salinity can adversely limit species diversity and their ecological niches.

To be specific, salinity is associated with negative osmotic potential which inhibits seed germination (Patel et al., 2010) and debilitates cell turgidity (Patel and Pandey, 2009). As a consequence, reduced plant cell elongation and photosynthetic area retards growth and, in some circumstances, plants fail to reproduce. Plants in sodic soils absorb excessive amounts of sodium (Na^+^) ions which replace potassium (K^+^) ions in plant biochemical functions (Roy and Chowdhury, 2020). Sodium toxicity stunts plants, suppresses net primary productivity, and deteriorates the nutritive value of forage crops (Temel et al., 2015; Worku et al., 2019). Irrigation measures that eliminate salts (Hanson et al., 2006) and soil amendments with gypsum and manure (Matosic et al., 2018) are traditionally used to manage soil salinity. These physical techniques involve relatively higher costs and unsustainable effects compared to the alternative of salt tolerant crop species (Fita et al., 2015).

**FIGURE 1:**
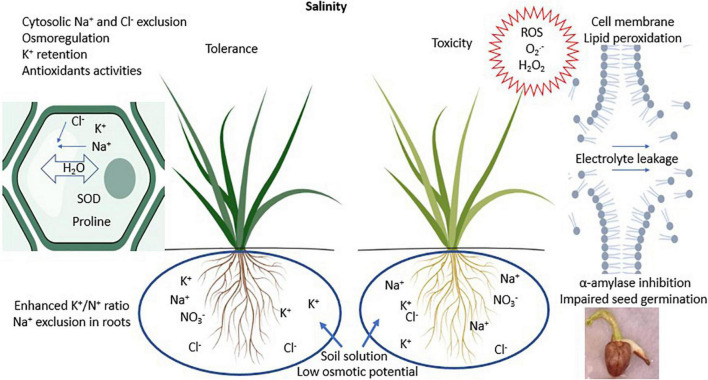
Schematic illustration of salinity effects to plants and its tolerance. SOD stands for superoxide dismutase; ROS stands for reactive oxygen species. Illustrations were created with BioRender.com.

Salt tolerant crop species are relatively convenient and affordable compared to physical measures used to manage soil salinity. The salinity tolerance in plants involves mechanisms of maintaining favorable water potential, balanced nutrient uptake, net photosynthesis, and alleviating toxic effects of Na^+^ and Cl^–^ ions (Lambers et al., 2008). These mechanisms are illustrated in Figure 1. The primary purpose of characterizing crop species is to develop salt tolerant varieties with superior yields and forage nutritive value. The need for synergies among species is increasing with the particular focus to formulate wholistic cropping systems that can foster and sustain environmental and production benefits. Contemporary forage cropping systems in non-saline conditions are renowned for their provisioning of forage, regulating soil health and hydrology, and supporting microbial and animal communities (Rodriguez et al., 2006). Dagar (2018) highlighted the possible ways to augmented forage cropping systems in saline environments by incorporating salinity tolerant halophytic species with agronomic value and unique abilities to alleviate salinity and salt toxicity.

Soil salinity management is a long-standing topic particularly focusing on food and forage crops. The increasing need for ecologically sound crop and livestock production has prompted efforts to mimic natural ecosystems in cultivated systems. There is a tendency to focus on individual plant species tolerance to salinity to meet imminent production objectives with little regard for ecosystems. One of the major gaps in the management of salinity is to identify and harness ecosystem services of plant species in developing cropping systems that can rehabilitate saline soils and, in the process, sustain forage productivity and ecosystem health. This prompts the need to understand (i) how consistent are mechanisms of tolerance to salinity among forage species in different habitats, (ii) do species converge in their salinity tolerance mechanisms, (iii) what are the possible synergies among species in their adaptation to saline soils, and (iv) how can mechanisms of species tolerance and synergies be used to design forage cropping systems adapted to saline soils. Hence this article reviews and reanalyzes published data to determine crop yield loss to soil salinity, species mechanisms of salinity tolerance and ecosystem services, and their potential synergies which can be used to design forage cropping systems for sustainable and resilient agroecosystems.

## Methods

### Conceptual Framework

**FIGURE 2:**
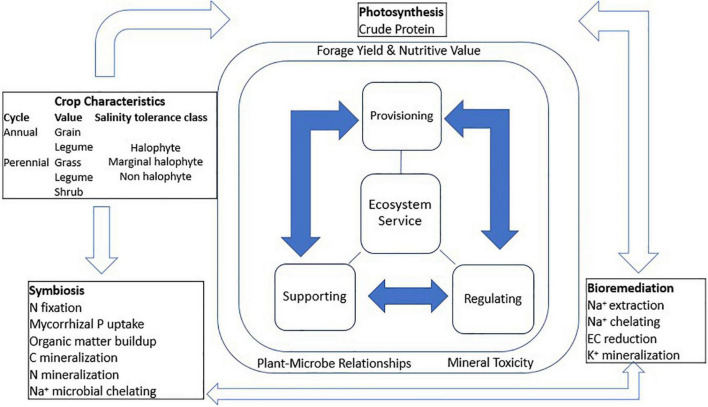
Flow diagram of conceptual framework for evaluating ecosystem services and synergies among forage crop species. Filled arrows represent interacting mechanisms and open arrows represent synergies.

This article provides a synthesis of correlations between salinity tolerance mechanisms and ecosystem services of forage crops. The first stage was to determine forage crop yield losses to soil salinity and discuss its tolerance. Thereafter, attempts were made to determine if forage crop species converge in their mechanisms of salinity tolerance and what this implies to forage productivity. In this regard, tolerance was considered the ability to maintain photosynthesis and dry matter yields with increasing salinity up to 200 mM NaCl concentrations in the propagation medium (Munns and Gillham, 2015). Salinity tolerance classes were according to the following: halophytes > 250 mM NaCl, marginal halophytes 100–250 mM NaCl, and non-halophytes < 100 mM NaCl (Munns et al., 2006). The data were obtained from 17 diverse experiments conducted under controlled greenhouse environments. The species were profiled according to crop duration, salinity tolerance classes, and their ecosystem services over a range of saline conditions (saturated soil extract EC > 4 dS m^–1^). Subsequently, crop species were categorized according to their ecosystem services reported in saline soils in arid and semi-arid habitats across continents. These involved studies spanning 1–5 years conducted in field conditions, each with a non-saline soil control besides an increasing magnitude of treatment to strongly saline soils. The criteria of Adhikari and Hartemink (2016) was used with some modifications to determine provisioning, regulating, and supporting ecosystem services of forage crops. Figure 2 shows a schematic association between crop categories and their association with ecosystem services, key functions, and synergies. Consequently, provisioning services included forage dry matter biomass yield and nutritive value. Forage crude protein was consistently reported along with soil salinity treatments in a number of studies; hence, this was chosen as a measure of provisioning services for forage nutritional value. Regulating ecosystem services included soil organic carbon and Na^+^ ion accumulation in crop biomass and their role in reducing soil salinity (bioremediation). Lastly, supporting services were crop microbe interactions that support nutrient cycling in forage cropping systems. The identified synergies were used as indicators to illustrate the model cropping systems that can alleviate soil salinity and enhance forage crop productivity and nutritive value.

### Data Acquisition and Analysis

The data for this review were obtained from the results of 20 studies published in peer-reviewed journals between the years 2000 and 2021. The literature search was done on the Google Scholar website. There is a sheer number of field investigations to identify physiological mechanisms of salinity tolerance because of their need for controlled environments. Hence, the relationship between salinity, photosynthesis and relative yield of seventeen crops in greenhouse studies was analyzed. Relative forage biomass was computed as the percentage forage accumulation of each crop in saline media against yield obtained in non-saline conditions. To determine crop ecosystem services, there were a total of 78 data entries representing crops evaluated in field trials whose details are recorded in Supplementary Table S1. Data was organized according to the location of study, crop name, cropping system (mono crop vs. polyculture), soil EC_e_ (saturated paste extract), and crop duration and cited authors. Additional variables including accumulated soil organic carbon, forage dry biomass, forage crude protein, soil EC change, and Na^+^ ion uptake. Data were subjected to the multivariate analysis procedure to determine correlations between variables and accordingly relationships among forage crop species. In doing so, the FactoMiner package was deployed to conduct principal component analysis (PCA) which also involved standardizing data according to the deviation of individual variates from the sample mean divided by sample standard deviation (R Core Team, 2019). The charts were visualized using the fviz_pca_biplot function in the R software version 3.6.3 (R Core Team, 2019).

## Results and Discussion

### Yield Loss Due to Soil Salinity and Salinity Tolerance

The yield losses of selected forage crop reported in saline soils were on average 5.4 Mg ha^–1^ and ranged from 1 Mg ha^–1^ in arid subtropical conditions to 22 Mg ha^–1^ in semi-arid tropical environments (Table 1). These values corresponded to increasing exchangeable sodium percentage (ESP) from 12 to 27. Salinity is adversely compounded with sodicity creating a disequilibrium favoring CaCO_3_ adsorption to cation exchange sites where Na^+^ ion is displaced and released in the soil solution (Wong et al., 2010). An overaccumulation of sodium greater than 15% of exchangeable cation has serious consequences of diminishing plant water and potassium uptake. Although this threshold varies with crop species, the ESP at which yield losses exceed 50% distinguishes crops that are intolerant compared to those that tolerate salinity (Pal et al., 2006).

**TABLE 1:** Forage crop losses to salinity in arid and semiarid environment.

Location	Habitat	Species	Classification	EC dS m^–1^	ESP (%)^†^	Change in yield (Mg ha^–1^)^‡^	References
Turkey	Arid	*Medicago sativa* L.	Non-halophyte	9.8	11.9	−3	Yalti and Aksu, 2019
		*Lotus corniculatus* L.	Non-halophyte	9.8	11.9	−3	
		*Onobrychis sativa* Lam.	Non-halophyte	9.8	11.9	−2	
		*Cynodon dactylon* (L.) Pers.	Non-halophyte	10.0	12.0	+ 1	Temel et al., 2015
		*Chloris gayana* Kunth.	Non-halophyte	10.0	12.0	+ 1	
		*Festuca arundinacea* Schreb.	Non-halophyte	10.0	12.0	−1	
Ethiopia	Semi arid	*Cenchrus ciliaris* L.	Non-halophyte	16.0	25.0	−7	Worku et al., 2019
		*Sorghum sudanense* (Piper) Stapf	Non-halophyte	10.0	21.0	−22	
		*Chloris gayana* Kunth.	Non-halophyte	18.0	27.0	−6	
Iran	Arid	*Sorghum bicolor* L. Moench	Non-halophyte	11.0	–	−3	Tabatabaei et al., 2012
		*Kochia scoparia* (L.) Schrad.	Halophyte	23.0	–	−1	Nabati et al., 2011
California	Arid	*Pennisetum purpureum* Schumach.	Non-halophyte	25.0	-	−7.8	Wang et al., 2002
Iran	Arid	*Sorghum bicolor* L. Moench	Non-halophyte	14	–	−10	Hedayati-Firoozabadi et al., 2020
		*Kochia scoparia* (L.) Schrad.	Halophyte	14	–	−4	
Iran	Arid	*Brassica napus L.*	Non-halophyte	10	–	−0.6	Shabani et al., 2013

*^†^ESP, Exchangeable sodium percentage.*

*^‡^Change in yield = forage yield in saline soils – forage yield in non-saline soils.*

The ways by which some forage species tolerate salinity are summarized in Figure 1 and Table 2. The C_4_ grass species *Aeluropus lagopoides* (L.) Thwaites and *Panicum antidotales* Retz. have been reported to retain similar shoot moisture with increasing salinity levels from 0 to 140 mM NaCl (Rafay et al., 2021). Maintenance of water balance in salt tolerant grasses is associated with the accumulation of organic osmolytes including proline and sugars which balance against negative osmotic pressure exerted by Na^+^ ions in the media around roots (Xu and Fujiyama, 2013). This phenomenon is also evident in the grass species *Imperata cylindrica* (L.) P. Beauv., *Eragrotis amabilis* (L.) Wight and Arn., *Cynodon dactylon* (L.) Pers., and *Digitaria ciliaris* (Retz.) Koeler (Roy and Chakraborty, 2017), *Festuca arundinacea* Schreb. and *Poa pratense* L. at 200 mM NaCl (Xu and Fujiyama, 2013), and *Agropyron desertorum* L. exposed to 150 mM NaCl (Sheikh-Mohamadi et al., 2017). Under increasing salinity, the dominance of Na^+^ uptake over K^+^ adversely affects plant metabolism (Lambers et al., 2008). Potassium plays vital roles in plant metabolism and growth including maintaining osmotic potential and activity of enzymes involved in protein and carbohydrate synthesis (Hasanuzzaman et al., 2018). Potassium also enhances tolerance to salinity stress for instance by reducing activity of reactive oxygen species and their toxicity. C_3_ species *Lathyrus sativus* L. and *Festuca arundinacea* Schreb. can maintain relatively constant shoot K^+^ ion concentrations in non-saline conditions up to 150 and 200 mM NaCl concentrations, respectively (Xu and Fujiyama, 2013; Tokarz et al., 2020). In this case, *Festuca arundinacea* Schreb. limited Na^+^ uptake and transport to the shoots (Xu and Fujiyama, 2013). In the leaves, halophytes suppress leakage of K^+^ ions to the apoplasm by maintaining membrane stability as observed in halophytic grass *Panicum antidotales* Retz. and *Aeluropus lagopoides* (L.) Thwaites grown in saline environments up to 140 mM NaCl (Rafay et al., 2021). In addition, at moderate salinity (100 mM NaCl), *Panicum antidotales* Retz. is reported to retain activities of rubisco carboxylase similar to non-saline conditions. More pronounced effects have been recorded in *Sporobolus spicatus* (Vahl) Kunth which enhanced photosynthesis up to 400 mM NaCl (Hamilton et al., 2001). Maintaining similar or greater net photosynthesis with increasing salinity confers halophytes with relatively more shoot biomass accumulation compared to glycophytes (Bose et al., 2013). Species avert direct toxicity of Na^+^ and Cl^–^ ions as well as their role in generating reactive oxygen species. This is exemplified in enhanced exudation of Na^+^ and Cl^–^ ions by *Sporobolus spicatus* (Vahl) Kunth at 400 mM NaCl (Hamilton et al., 2001). *Festuca arundinacea* Schreb. and *Poa pratense* L. generate more antioxidant enzyme superoxide dismutase in response to 200 mM NaCl (Xu and Fujiyama, 2013), which degrade reactive oxygen species from photosystems I and II into less toxic H_2_O_2_ and O_2_ (Gill and Tuteja, 2010). These mechanisms represent standalone species characterization which, if harnessed in cropping systems, can alleviate impacts of soil salinity to crops and the environment.

**TABLE 2:** Mechanisms of salinity tolerance in forage species.

Enhanced mechanism	Salinity stress	Species	References
Water retention in shoots	140 mM NaCl	*Aeluropus lagopoides* (L.) Thwaites	Rafay et al., 2021
		*Panicum antidotales* Retz.	
Retention of K^+^ ions	150 mM NaCl	*Lathyrus sativus* L.	Tokarz et al., 2020
	200 mM NaCl	*Festuca arundinacea* Schreb.	Xu and Fujiyama, 2013
Membrane stability	140 mM NaCl	*Aeluropus lagopoides* (L.) Thwaites	Rafay et al., 2021
		*P. antidotales* Retz.	
Rubisco activity	100 mM NaCl	*P. antidotales* Retz.	Hussain et al., 2020
Photosynthesis	400 mM NaCl	*Sporobolus spicatus* (Vahl) Kunth	Hamilton et al., 2001
Proline and soluble sugar content	200 mM NaCl	*Imperata cylindrica* (L.) P. Beauv	Roy and Chakraborty, 2017
		*Eragrotis amabilis* (L.) Wight and Arn	
		*Cynodon dactylon* (L.) Pers.	
		*Digitaria ciliaris* (Retz.) Koeler	
		*Festuca arundinacea* Schreb.	Xu and Fujiyama, 2013
		*Poa pratense* L.	
	150 mM NaCl	*Agropyron desertorum* L.	Sheikh-Mohamadi et al., 2017
Increased activity of SOD	200 mM NaCl	*Festuca arundinacea* Schreb.	Xu and Fujiyama, 2013
		*Poa pratense* L.	
Na^+^ and Cl^–^ ion exudation	400 mM NaCl	*Sporobolus spicatus* (Vahl) Kunth	Hamilton et al., 2001

### Implications of Species Salinity Tolerance Mechanisms to Forage Productivity

**FIGURE 3:**
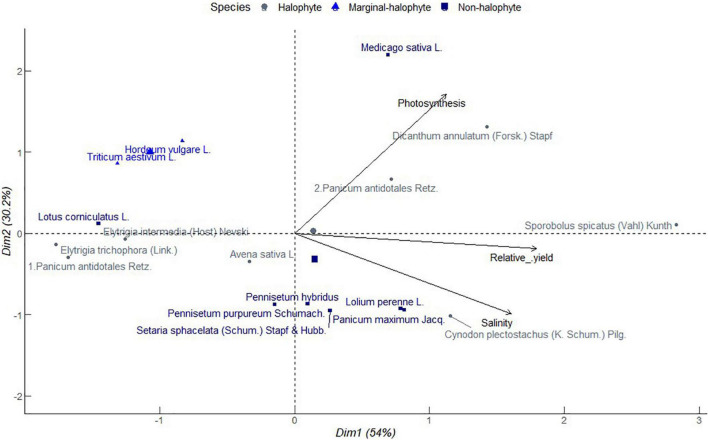
Principal component analysis of relationship between soil salinity, photosynthesis, and relative biomass for different forage crop species obtained from different studies conducted in greenhouse environments from 2000 to 2021. Salinity tolerance class is in the order of halophytes > 250 mM NaCl, marginal halophytes 100–250 mM NaCl and non-halophytes < 100 mM NaCl (Munns et al., 2006).

It is of primary significance that grass and forb species mechanisms of salinity tolerance translate to sustainable forage biomass production and resilience to further exposure to salt stress. Figure 3 shows a PCA of relative forage biomass of popular annual and perennial grass and legume species from different studies. Principle component 1 explained 54% of total variation associated with increasing photosynthesis, and relative biomass as salinity increased in the propagation media. Salinity was strongly correlated with relative yield, but no correlation with photosynthesis. It indicates improving forage yield is possible through improving photosynthetic capacity in those species or selecting photosynthetically efficient species. Star grass (*Cynodon plectostachyus* (K. Schum.) Pilg.), a halophytic grass, clustered together with non-halophytes including perennial ryegrass (*Lolium perenne* L.) and guinea grass (*Panicum maximum* Jacq.) and less proximately with *Setaria sphacelata* (Schum.) Stapf and Hubb. and *Pennisetum hybridus*, all of which had close associations with increasing soil salinity. Alfalfa which is a non-halophyte clustered with halophytes namely marvel grass (*Dicanthum annulatum* (Forssk.) Stapf) and blue panicum grass (*Panicum antidotales* Retz.) along increasing photosynthesis. Saltgrass (*Sporobolus spicatus* (Vahl) Kunth) which is a halophyte, stood alone with strong and near equitable contribution to increasing relative yield, photosynthesis and salinity. Saltgrass is reported to restrict the Na^+^ uptake with increasing soil salinity and secrete excess salts at night (Ramadan, 2001). This indicates the resilience of saltgrass to salinity, which is a potential attribute for sustaining net primary production in companion cropping. Birdsfoot trefoil (*Lotus corniculatus* L.), a non-halophytic legume, congregated with halophytic grasses including intermediate wheatgrass [*Elytrigia intermedia* (Host) Nevski, and *Elytrigia trichophora* (Link.)], and blue panicum grass (*Panicum antidotales* Retz.) associated with reducing salinity, photosynthesis, and relative yield. Intermediate wheatgrass has C_3_ photosynthetic machinery (Jaikumar et al., 2016) and is likely to coexist with birdsfoot trefoil unlike blue panicum a vigorous C_4_ grass. In this category, there was as well a cluster of barley (*Hordeum vulgare* L.) and wheat (*Triticum aestivum* L.) which are considered marginal halophytes. These annual grain crops are commonly diversified with annual legumes in time or space. Nevertheless, their proximal grouping with birdsfoot trefoil suggests a potential coexistence, for instance, in living mulch or in prolonged rotations. Living mulch refers to maintaining a perennial legume crop in an annual cropping system. Oat (*Avena sativa* L.), a halophytic annual grain, and *Pennisetum purpureum* Schumach., a non-halophytic grass, had near neutral contribution to salinity, photosynthesis, and relative yields hence potential candidates for long perennial annual rotations. Overall, the results of PC indicate a convergence of halophytes and non-halophytes in their physiology and yield response to salinity. This suggests possibilities for synchrony of diverse species functional groups in their adaptation to salinity stress which can support synergies that enhance overall forage productivity. In broad sense, such coexistence and facilitation can compensate for generally low forage productivity of halophytes as Dagar (2005) reports.

### Synergies Among Forage Crop Species in the Context of Soil Salinity

Synergies refer to the simultaneous increase or decrease in the provisioning of ecosystem services (Rodriguez et al., 2006). Forage crops are renowned for a wide range of ecosystem services. Among these, provisioning, regulating, and supporting services have been amplified in an effort to address escalating adversities of climate change, environmental degradation, and low agricultural productivity. In cultivated, non-saline conditions, perennial forage crops have gained attention due to their ability to reverse soil degradation. Perennial grasses are renowned for below-ground carbon sequestration, while legumes support nitrogen accretion in the agroecosystems. With this background, plants support microbial diversity with feedback to nutrient cycling, net primary production and sustainable ecosystems. These crops regulate nutrient cycling, soil moisture retention, soil erosion while supporting the accretion of soil carbon and nitrogen.

Overboard, crop diversification to mimic the stability of primary production and resilience of natural vegetation to disturbances is increasingly appealing to the design of sustainable agroecosystems (Islam and Ashilenje, 2018). For example, recently, grass-legume mixtures maintained without fertilizers have been demonstrated to enhance forage yield and nitrogen and crude protein concentrations, and to buffer against nitrous oxide emissions in non-saline soils (Ashilenje, 2018). Islam and Ashilenje (2018) have discussed the benefits of diversified cropping systems in detail. In summary, mixtures assemble legumes and grasses that scavenge for excess soil mineral N, which, if left, serves as the substrate for denitrification. Accumulated mineral N inhibits nitrogenase enzyme activities of N fixation. Grasses amass carbon in their expansive root system and in the process alleviate losses through mineralization. These qualities of grasses recondition the soil in a gradual process that balances nutrient turnover from atmospheric nitrogen fixation, mineralization, and organic matter input from legume crops in mixtures. As a consequence, forage yields and soil health can be sustained while forfeiting shifts in ecosystem balance associated with fertilizers, tillage, and related anthropogenic factors. Intensified forage production on cultivated lands can support livestock health on less land resources unlike extensive systems associated with overgrazing and consequently, loss of plant canopy cover (Rutherford and Powrie, 2013). There are indicators that the agronomic and ecological benefits of diversified forage crop systems with halophytes can ameliorate soil salinity (Ghaffarian et al., 2021). In this light, ecosystem services reported in diverse forage crops growing in saline soils with EC ranging from 4.4 dS m^–1^ (moderately saline) to 41 dS m^–1^ (extremely saline) are analyzed in sections “Provisioning Ecosystem Services of Forage Production” to “Supporting Ecosystem Services for Soil and Animal Health.”

#### Provisioning Ecosystem Services of Forage Production

**FIGURE 4:**
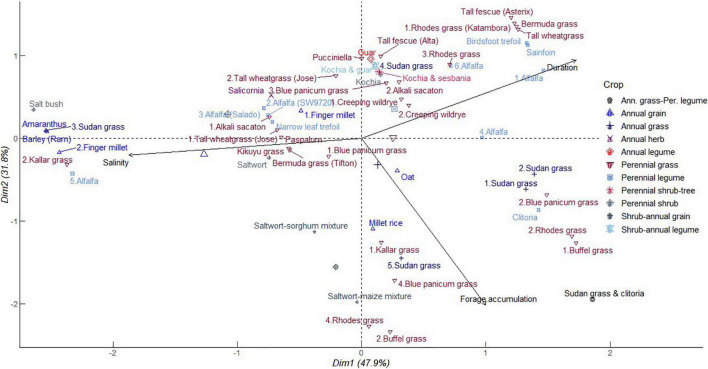
Principal component analysis of dry biomass accumulation of different forage crops in saline conditions. The data points were obtained from seventeen different studies published from 2000 to 2021. Crops are numbered differently according to study and labels in parenthesis represent crop variety.

As shown in Figure 4, PC1 explained 47.9% of the total variation, which was positively correlated with high soil salinity and negatively so with forage biomass accumulation and duration of production. Annual forage grain monocrops including barley, finger millet, and Sudan grass clustered with alfalfa and kallar grass in close association with increasing soil salinity but decreasing forage biomass accumulation. At moderate soil salinity, perennial grasses including tall wheatgrass, blue panicum grass, paspalum, Bermuda grass, alkali sacaton, and kikuyu grass, grouped together with alfalfa and narrow leaf trefoil. Crops in this category, with exception of alfalfa and kikuyu grass, exhibit tolerance to salinity and indicate a wide variety of options for diversification against effects of soil salinity (Guo et al., 2014; Xiang et al., 2015). This cluster also included saltwort and salicornia which are eminent halophytes. On the other side, Sudan grass tied together with buffel grass, Rhodes grass, blue panicum grass, kallar grass, and millet rice in a cluster contributing to increasing forage accumulation. Sudan grass also formed a distinct group with Rhodes grass, buffel grass, and blue panicum grass which had intermediate contributions to forage accumulation and crop duration in PC2 (31.8%). Alfalfa again congregated with perennial legumes namely birdsfoot trefoil and sainfoin as well as Bermuda grass, tall wheat grass, tall fescue, and Rhodes grass associated with extended cropping period but decreasing forage accumulation and soil salinity. It is worthy to note that Sudan grass crop and alfalfa have an unrestricted pattern ranging from short duration of growth in high soil salinity with low yields and the inverse of high forage biomass at reducing salinity. Sudan grass-clitoria mixture had a profound contribution to forage biomass accumulation (Abusuwar, 2019). This implies that ineptness of annual species to saline conditions can be leveraged by mixtures. In annual cropping systems, adverse effects of soil salinity might be encouraged by water evaporation direct deposit of salts on the soil surface (Gabriel et al., 2012). This can be reversed by high crop density and retention of crop residues beyond one cropping season (Cuevas et al., 2019). Alfalfa and kallar grass displayed a wide range of influences from strong to negative correlation with soil salinity in a gradient of 6–41 dS m^–1^. Blue panicum grass and Rhodes grass followed a similar pattern.

The ubiquity of grass and alfalfa indicates that these species can coexist and buffer against limitations of soil salinity to forage biomass accumulation. More specifically, a combination of species selective preference for uptake of K^+^ over Na^+^ ions in grass (Guo et al., 2014) and narrow leaf trefoil (Teakle et al., 2006) and the abilities of halophytic grass to alleviate salt accumulation in soil can avert stresses to legumes susceptible to salinity. Hence, these relationships can sustain overall crop ecosystem primary production. These associations can be exploited across a gradient of soil salinity up to extreme levels as revealed in the results of PCA. Alfalfa, blue panicum grass, and Rhodes grass distribution varied from longer growth period to shorter growth span associated with increasing forage accumulation. This indicates that the provisioning services of these perennial forage crops are not tied to the duration of growth. This trend further explains the potential to enhance forage biomass accumulation of the perennial grasses during the early years of establishment or complementarity in mixtures with perennial legumes beyond establishment. Halophytic crops including Salicornia, saltwort, and kochia tended to be neutral in their association with forage biomass accumulation and soil salinity. The same applied to saltwort-maize, saltwort-sorghum, kochia-guar, kochia-sesbania, and kochia-sesbania-guar mixtures. These results again suggest a facilitating role of halophytes to perpetuate growth of annuals normally susceptible to salinity. The characteristic ability of halophytes to extract Na^+^ ions from soils, hence regulate salinity, are discussed in detail in section “Regulatory Ecosystem Services Against Soil Nutrient Imbalance and Phytotoxicity.”

#### Provisioning Ecosystem Services of Forage Nutritive Value

**FIGURE 5:**
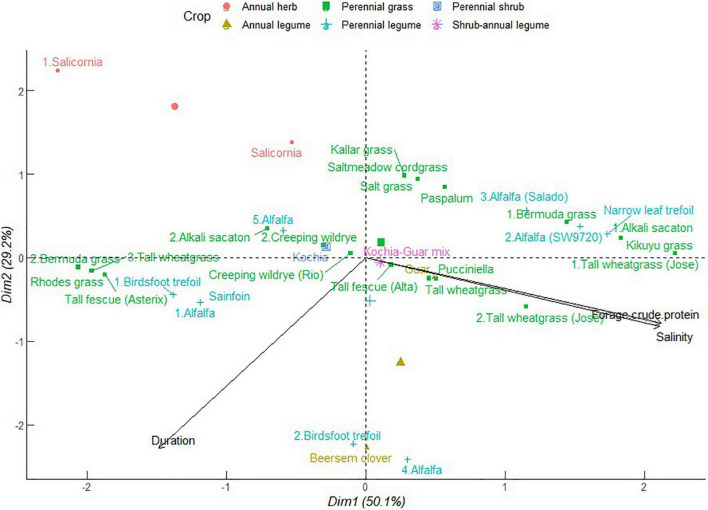
Principal component analysis of forage crude protein of different forage crops in saline conditions. The data points were obtained from seventeen different studies published from 2000 to 2021. Crops are numbered differently according to study and labels in parenthesis represent crop variety.

Together with yield, forage nutritive value determines animal health and production. The multivariate analysis revealed potential synergies among crops in their influence on forage crude protein in saline soils. As shown in Figure 5, PC1 explains 50.1% of the total variance. In this case, forage crude protein is correlated positively to soil salinity and negatively to crop duration. Tall wheatgrass, Bermuda grass, alkali sacaton, kikuyu grass, alfalfa, and narrow leaf trefoil contributed strongly in the cluster with increasing salinity and crude protein. This confounds the detriments of increasing soil salinity on nitrogen uptake and accumulation of proteins in forages as explained in the previous studies (Kumar et al., 2018; Kamran et al., 2020). Conversely, there are possible explanations supporting forage crude protein—soil salinity dependence. Certain amino acids may have osmoregulatory functions that alleviate the deleterious effects of Na^+^ ions on plant proteins (Kamran et al., 2020). Alfalfa, Bermuda grass, and tall wheatgrass exhibited indeterminate patterns that extend from association with increasing salinity and forage crude protein to a counteractive effect when they grow for longer periods. This group also comprised Rhodes grass, tall fescue, sainfoin, and birdsfoot. Alfalfa, birdsfoot trefoil, and berseem clover formed a cluster that balances between duration, salinity, and forage crude protein. Forage kochia and creeping wildrye appeared intermediary with respect to duration, soil salinity, and forage crude protein. These crops are extremely tolerant to salinity (Suyama et al., 2007; Waldron et al., 2020). These results unbind the traditional profiling of the compatibility of common forage crops and identify commonalities in species traits that can overcome temporal effects of soil salinity to forage productivity and nutritive value.

Although not included in this analysis, grasses tend to accumulate more neutral detergent fiber (NDF) and lower crude protein than legumes. As depicted here, grasses are versatile in their range of crude protein concentration, some of which march that of legumes. A combination of grasses and forbs to generate forage of NDF ≤ 50% and crude protein ≥ 20% of dry matter is recommended to support animal and ruminal microbial energy needs which invigorate animal health and reproduction (Collins and Fritz, 2003). The balance of fiber and crude protein in crop residues also has implications on carbon-to-nitrogen ratio which influences soil microbial communities and their functions. These functions are discussed in detail in supporting ecosystem services in section “Supporting Ecosystem Services for Soil and Animal Health.”

#### Regulatory Ecosystem Services Against Soil Nutrient Imbalance and Phytotoxicity

**FIGURE 6:**
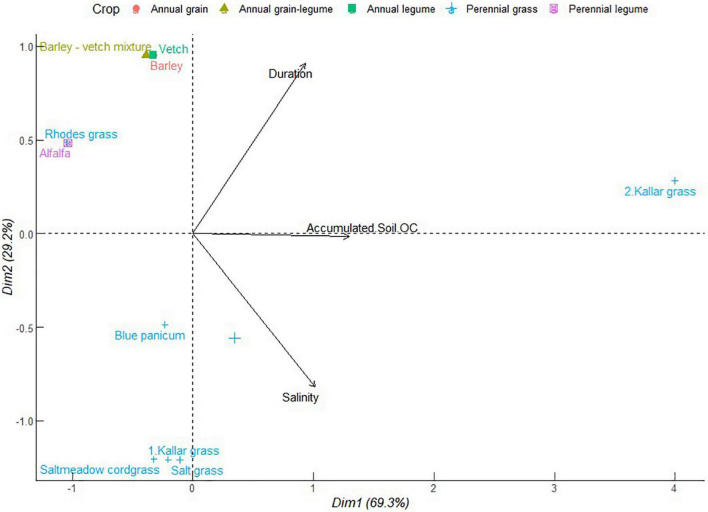
Principal component analysis of accumulated soil organic carbon (OC) of different forage crops in saline conditions. The data points were obtained from four different studies published from 2000 to 2021. Crops are numbered differently according to study.

As depicted in Figure 6, PC1 represented 69.3% of total variation with the positive correlation between accumulated soil organic carbon, soil salinity, and duration of cropping systems. This cluster had the sole contribution of one of the kallar grass crops. However, accumulated soil organic carbon was independent of the role of soil salinity or crop duration. The majority of the crops were clustered in the zone of declining contribution of duration, soil organic carbon, and salinity. It is less probable that crops adapted to salinity also promote soil organic carbon accumulation. This may be explained by depressed primary production and carbon sequestration (Lal, 2009). The reverse process of alleviating sodicity can help resuscitate plant growth in the process to improve soil carbon sequestration. A combination of species mechanisms and crop combinations that generate crop residues can help boost soil surface cover and soil organic matter accumulation. As a consequence, soil organic matter ameliorates against salinity primarily *via* improved soil structure and therefore enhanced infiltration of Na^+^ ions down the soil profile (Bhuiyan et al., 2016). The confluence of soil carbon accumulation and salinity reduction have been demonstrated in tree-understory halophytic grass silvipastoral systems (Singh et al., 2022). The successful soil amelioration in this case was attributed to enhanced uptake of Na^+^ ion by understory grass whose forage accumulation was facilitated by enhanced regime of photosynthetic active radiation, as influenced by the companion perennial tree canopy. Other possible mechanisms include organic matter decomposition and subsequent release of humic acids which can sequester Na^+^ ions and help to reduce soil sodium exchangeable potential and electrical conductivity (Bhuiyan et al., 2016).

**FIGURE 7:**
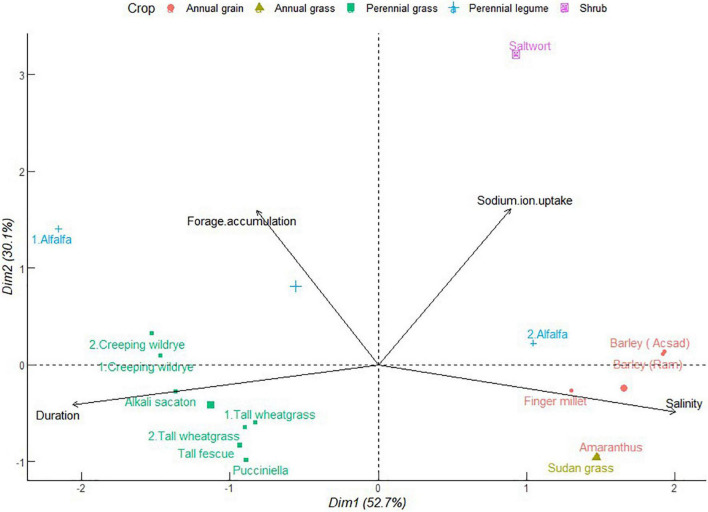
Principal component analysis of sodium (Na^+^) ion uptake of different forage crops in saline conditions. The data points were obtained from three different studies published from 2000 to 2021. Crops are numbered differently according to study and labels in parenthesis represent crop variety.

There is growing interest in the bioremediation of saline soils to sustain forage production. For example, Rabhi et al. (2010) determined that sea purslane (*Sesuvium portulacastrum* L.), a halophyte, can extract an estimated 1 ton of Na^+^ ions ha^–1^ year^–1^. This subsequently enhanced plant water and K^+^ ion retention together with shoot biomass in succeeding barley (*Hordeum vulgare* L.). Saltwort has similar potential, for instance, accumulating up to 125 g of Na^+^ ion kg^–1^ of dry matter within a span of 1 year (Toderich et al., 2008). In Figure 7, PC1 explained 52.7% of total variation strongly associated with increasing salinity as crop duration decreases. Salinity clustered tightly with barley, finger millet, amaranthus, Sudan grass, and alfalfa. Conversely, creeping wildrye, alkali sacaton, tall wheatgrass, tall fescue, and pucciniella clustered together along the increasing crop duration but decreasing salinity. In PC2 which explained 30.1% of the total variation, forage accumulation was correlated with sodium uptake profoundly associated with saltwort and to a lesser extend alfalfa. Enhanced shoot biomass and Na^+^ ion accumulation are jointly classic qualities required for efficient bioremediation of saline soils by halophytes (Qadir et al., 2007). Nevertheless, species compatibility, forage value, and temporal effects of halophytic species are factors that count in the approach to integrate bioremediation in salt-affected cropping systems. The ability of common forage crops to reduce soil salinity is recorded in Table 3. Kallar grass is reported to have the highest influence of reducing soil salinity by 4 and 5 dS m^–1^ annually (Akhter et al., 2004; Tawfik et al., 2013). This behavior is associated with altering of soil structure to support deep leaching of Na^+^ ions (Akhter et al., 2004). Saltgrass (4 dS m^–1^), saltmeadow cordgrass (3 dS m^–1^), and blue panicum grass (2 dS m^–1^) are reported to have intermediate effects (Tawfik et al., 2013; Gelaye et al., 2019). Rhodes grass, Sudan grass, and alfalfa rank on the lower range, each reducing the soil salinity by 1 dS m^–1^ year^–1^ (Gelaye et al., 2019). Unlike physical amendments to soil salinity, plant-based mechanisms of reducing salinity have inconsistent effects. Hence, the need for species- and site-specific determinations of optimum cropping systems to alleviate salinity.

**TABLE 3:** Ability of common forage crops to reduce soil salinity.

Crop		Soil Salinity			References
	Period	Initial	Change during entire period	Annual change	
			
	Years	dS m^–1^			
Blue panicum	2	14	−4	−2	Gelaye et al., 2019
Alfalfa	2	6	−1	−1	
Sudan grass	2	14	−1	−1	
Rhodes grass	2	6	−1	−1	
Kallar grass	1	15	−5	−5	Tawfik et al., 2013
Salt grass	1	15	−4	−4	
Saltmeadow cordgrass	1	15	−3	−3	
Kallar grass	5	22	−20	−4	Akhter et al., 2004

#### Supporting Ecosystem Services for Soil and Animal Health

Discussions of plant microbe interactions and their supporting ecosystem services are presented in this section. Biological nitrogen fixation, phosphorus solubilization and uptake, and nitrogen mineralization are important in this respect. Soil salinity has antagonistic mechanisms against mineral N, but a mixture of positive and negative influences on P uptake depending on plant species (Grattan and Grieve, 1999). Salinity can also suppress the availability of these minerals in the soils. Elevated levels of Cl^–^ ions act against NO_3_^–1^ uptake and activity of nitrate reductase enzyme (Farissi et al., 2014). Increasing salinity also depresses N mineralization (Lodhi et al., 2009). Increasing salinity has particularly been associated with reducing P uptake in alfalfa (Farissi et al., 2014) and explained by the low solubility of P-Ca complexes (Grattan and Grieve, 1999). These important functions of crop N and P nutrition are mediated by soil microbes which are devastated by soil salinity (Kizildag et al., 2013). This challenge has traditionally been addressed by symbiosis between crops and salinity tolerant microorganisms. There are limited studies quantifying the N_2_ fixation by forage crops in saline conditions. Among the few studies of this nature, Bruning et al. (2015) reported no significant effect of increasing soil salinity (1.7–16 dS m^–1^) on biological N fixation by sweet clover (∼100%) and alfalfa (90–70%) except at 20 dS m^–1^ (80 and 43%, respectively). It is suggested that N fixing bacteria release indole acetic acid at levels that safeguard the symbiotic process and plant growth from severe levels of soil salinity (William and Signer, 1990). This mechanism is pertinent to genus *Sinorhizobium* (Rejii and Mahdhi, 2014). The question arising is how these benefits can be maintained in cropping systems under saline conditions. The understanding of species diversity and their interactions with microbial communities in saline soils can help discern related ecosystem support services. Drawing a parallel from non-saline conditions, grass-legume mixtures can stimulate greater N2 fixation compared to legume monocrops and enrich grass crude protein compared to grass monoculture (Ashilenje, 2018). Nitrogen-fixing bacteria are more salt tolerant to soil salinity than their hosts (Zahran, 1999). This characteristic of plant growth promoting bacteria coupled with host species interactions that deescalate soil salinity and toxicity can potentially create environments for enhanced symbiosis in forage cropping systems. Soil microbe-mediated P uptake contrasts that of N. For instance, the role of mycorrhizae in augmenting P uptake and growth of grasses can be jeopardized by poor colonization of hosts as soil salinity increases (Pedranzani et al., 2016; Romero-Munar et al., 2019). Similarly, Arbuscular mycorrhiza fungi (AMF) have been observed to alleviate moderate effects of salinity on P uptake, photosynthesis, and plant growth in berseem clover and alfalfa (Shokri and Maadi, 2009; Shi-Chu et al., 2019). The role of AMF in alleviating the effect of salinity can be upmodulated in forage legumes. For example, co-inoculation with rhizobium can enhance AMF colonization of alfalfa roots (Ashrafi and Zahedi, 2014). As a consequence, these microbes interact to step-up N_2_ fixation, P uptake, photosynthesis, and shoot biomass yields.

#### Designing Appropriate Forage Cropping Systems for Saline Soils

**FIGURE 8:**
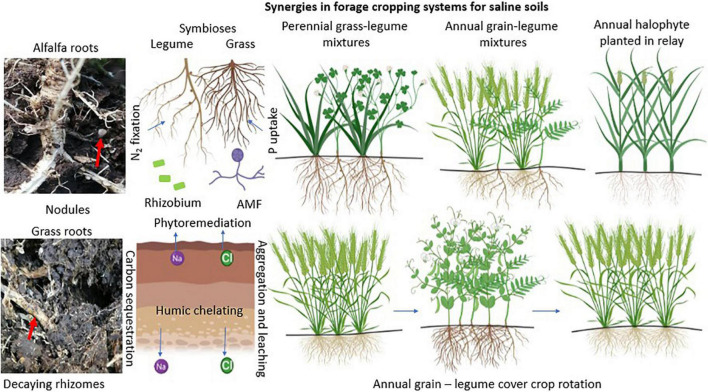
Summary of plant-microbe synergies relevant for forage cropping systems designed to adapt to saline conditions. AMF stands for arbuscular mycorrhizal fungi. Illustrations were created with BioRender.com.

All of the above information showed that there is overwhelming evidence that soil microbes are ingredients of ecosystem sustainability by virtue of their roles in soil nutrient cycling. Soil microbial communities rely on energy from their plant hosts, and when established, they can revitalize crops against the effects of soil salinity and support forage productivity and nutritive value. This potential has not been fully exploited in orchestrated cropping systems to alleviate soil salinity and its effects on ecosystem health. Figure 8 provides a synthesis of plant-microbe synergies that can be perpetuated in cropping systems designed to alleviate the effect of salinity on forages and sustain forage production and resilience. Annual and perennial cropping systems are depicted, premised on potential complementarities and symbioses. There are four scenarios with implications for designing forage cropping systems to exploit ecosystem services identified in this study. The first one is a perennial grass-legume mixture lasting for at least 3 years with an initial inoculum of nitrogen-fixing bacteria and AMF. This system might include a halophytic grass that extracts significant amounts of Na^+^ ions from soils and allows legumes to thrive while entrenching microbial N and P uptakes. The second scenario is an annual grain legume mixture with the grain crop designed to reduce Na^+^ ion contents in soils with repeated planting every year. Again, legumes in this case need to have the capacity to fix substantial amounts of N within the growing season. The third scenario is establishing annual halophytes in relay (before harvesting) with annual grain-legume mixtures in a sequence that extends through fallow periods. The halophyte is designed not only to adapt in marginal weather conditions but also able to absorb Na^+^ ions and supply surface mulch for the next annual grain-legume crop mixture. The fourth system is an annual grain monocrop-legume monocrop in rotation. This system advances the benefits of optimum forage productivity and cover crops over at least 3 years of rotation. Overall, the cropping systems suggested here are designed to exploit the ecosystem services that enhance N and P supplies and optimize forage productivity and nutritive value. These systems are also developed to sequester carbon and alleviate salt buildup and toxicity in a gradual process that leads to sustainable forage production in areas prone to salinity.

## Conclusion

This article explored species mechanisms and synergies that can be incorporated in cropping systems to alleviate the challenges of soil salinity and sustain forage productivity in arid regions. It is clear that halophytic and non-halophytic forage crop species have convergent mechanisms of salinity tolerance manifested in enhanced photosynthesis and productivity. There are indicators of ubiquity of ecosystem services of both halophyte and non-halophytic species along a continuum of increasing soil salinity. Potential grass-forb, annual-perennial, halophytes-non-halophytes, and plant-bacteria-fungi synergies against effects of soil salinity were identified. These synergies can be harnessed in designing sustainable forage cropping systems that ameliorate saline soils and improve nutrient cycling to sustain optimum forage productivity.

## Data Availability Statement

The original contributions presented in the study are included in the article/Supplementary Material, further inquiries can be directed to the corresponding author.

## Author Contributions

DA and AN conceived and designed the study. AN supervised the work. DA, EA, AH, and AE collected and analyzed the data. DA drafted the manuscript. AH, LK, KD, and AN validated the data analysis and reviewed all the versions of the manuscript. All authors contributed to the article and approved the submitted version.

## Conflict of Interest

The authors declare that the research was conducted in the absence of any commercial or financial relationships that could be construed as a potential conflict of interest.

## Publisher’s Note

All claims expressed in this article are solely those of the authors and do not necessarily represent those of their affiliated organizations, or those of the publisher, the editors and the reviewers. Any product that may be evaluated in this article, or claim that may be made by its manufacturer, is not guaranteed or endorsed by the publisher.
