# Supplementary material for: Crop Species Mechanisms and Ecosystem Services for Sustainable Forage Cropping Systems in Salt-Affected Arid Regions
Source: Front Plant Sci. 2022 May 24;13:899926. doi: 10.3389/fpls.2022.899926 (PMC9171386; doi:10.3389/fpls.2022.899926)
Supplement: Supplementary file 1 [file Table_1.DOCX]

Supplementary Material

# Table S1. Supplementary data representing influence of forage crops growing for diverse durations on soil properties and forage productivity in arid and semi-arid areas

| **Location** | **Crop** | **Cropping System** | **Soil EC**† | **Period (Years)** | **Accumulated Soil OC (g/kg)** ‡ | **Accumulated Forage (Mg ha^-1^)** | **CP (%)**§ | **Change in EC**† | **Na uptake (g/kg)** | **Reference** |
| --- | --- | --- | --- | --- | --- | --- | --- | --- | --- | --- |
| Igdir-Turkey | Alfalfa | Monocrop | 9.8 | 3 |  | 12.8 | 15.8 |  |  | Temel et al., 2016 |
|  | Birdsfoot trefoil | Monocrop | 9.8 | 3 |  | 8.8 | 14 |  |  |  |
|  | Sainfoin | Monocrop | 9.8 | 3 |  | 9.1 | 15.5 |  |  |  |
|  | Kochia | Monocrop | 14 | 2 |  | 6.4 | 11.9 |  |  | Ghaffarian et al., 2021 |
|  | Sesbania | Monocrop | 14 | 2 |  | 2.6 | 28.2 |  |  |  |
|  | Guar | Monocrop | 14 | 2 |  | 4.1 | 20.3 |  |  |  |
|  | Kochia-Sesbania mixture | Mixture | 14 | 2 |  | 6 | 16.7 |  |  |  |
|  | Kochia-Guar mixture | Mixture | 14 | 2 |  | 5.1 | 16.4 |  |  |  |
|  | Sesbania-Guar mixture | Mixture | 14 | 2 |  | 4.4 | 23 |  |  |  |
|  | Kochia-Sesbania-Guar mixtures | Mixture | 14 | 2 |  | 6.3 | 18.8 |  |  |  |
| Riverside-California | Bermuda grass | Monocrop | 15 | 1 |  | 10.7 | 23.2 |  |  | Grattan et al, 2004 |
|  | Kikuyu grass | Monocrop | 15 | 1 |  | 10.5 | 26.5 |  |  |  |
|  | Paspalum | Monocrop | 15 | 1 |  | 8.8 | 13.6 |  |  |  |
|  | Tall wheatgrass | Monocrop | 15 | 1 |  | 7.8 | 30.6 |  |  |  |
|  | Narrow leaf trefoil | Monocrop | 15 | 1 |  | 6.6 | 26.3 |  |  |  |
|  | Alkali sacaton | Monocrop | 15 | 1 |  | 5.9 | 26.9 |  |  |  |
|  | Alfalfa cv SW9720 | Monocrop | 15 | 1 |  | 5.6 | 23.9 |  |  |  |
|  | Alfalfa cv Salado | Monocrop | 15 | 1 |  | 4.6 | 20.4 |  |  |  |
| Isfahan-Iran | Blue panicum | Monocop | 11 | 1 |  | 12 | nd |  |  | Eshghizadeh et al., 2012 |
| Hada-Alsham - Saudi Arabia | Sudan grass | Monocop | 5.5 | 2 |  | 24 | nd |  |  | Abusuwar, 2019 |
|  | Clitoria | Monocop | 5.5 | 2 |  | 27 | nd |  |  |  |
|  | Sudan grass- Clitoria mixture | Mixture | 5.5 | 2 |  | 40 | nd |  |  |  |
| Igdir-Turkey | Tall fescue | Monocrop | 9.8 | 3 |  | 5.1 | 9.1 |  |  | Temel et al., 2015 |
|  | Bermuda grass | Monocrop | 9.8 | 3 |  | 6.4 | 7.1 |  |  |  |
|  | Rhodes grass | Monocrop | 9.8 | 3 |  | 5.9 | 8.2 |  |  |  |
|  | Tall wheatgrass | Monocrop | 9.8 | 3 |  | 6.8 | 8 |  |  |  |
| Addis Ababa-Ethiopia | Buffel grass | Monocrop | 17.9 | 3 |  | 37 |  |  |  | Qureshi et al., 2018 |
|  | Blue panicum | Monocrop | 17.9 | 3 |  | 30 |  |  |  |  |
|  | Sudan grass | Monocrop | 17.9 | 3 |  | 27 |  |  |  |  |
|  | Rhodes grass | Monocrop | 17.9 | 3 |  | 36 |  |  |  |  |
| Adana-Turkey | Birdsfoot trefoil | Monocrop | 16 | 4 |  |  | 18.9 |  |  | Boga et al., 2014 |
|  | Beersem clover | Monocrop | 16 | 4 |  |  | 20.3 |  |  |  |
|  | Alfalfa | Monocrop | 16 | 4 |  |  | 22.6 |  |  |  |
|  | Barley | Monocrop | 6.9 | 3 | 0.27 |  |  |  |  | Mitchel et al., 2000 |
|  | Vetch | Monocrop | 6.9 | 3 | 0.27 |  |  |  |  |  |
|  | Barley - vetch mixture | Mixture | 6.9 | 3 | 0.21 |  |  |  |  |  |
| Fresno-California | Alfalfa cv 'salado' | Monocrop | 6.9 | 2 |  | 16.3 | 23.7 |  | 5.3 | Suyama et al., 2007 |
|  | Tall wheatgrass cv 'Jose' | Monocrop | 19.1 | 2 |  | 6 | 15.6 |  | 8.9 |  |
|  | Creeping wildrye | Monocrop | 13.3 | 2 |  | 10 | 16.4 |  | 1.2 |  |
|  | Tall wheatgrass | Monocrop | 17.6 | 2 |  | 6.3 | 11.2 |  | 6.4 |  |
|  | Creeping wildrye | Monocrop | 12.9 | 2 |  | 11 | 13.9 |  | 2.5 |  |
|  | Pucciniella | Monocrop | 15 | 2 |  | 3.9 | 17.7 |  | 6 |  |
|  | Tall fescue | Monocrop | 12.7 | 2 |  | 3.8 | 19 |  | 11.5 |  |
|  | Alkali sacaton | Monocrop | 12.4 | 2 |  | 7.6 | 12.1 |  | 1.3 |  |
| Kyzylkum-Uzbekistan | Saltwort | Monocrop | 17.8 | 1 |  | 11.5 |  |  | 125 | Toderich et al., 2008 |
|  | Saltwort-maize mixture | Mixture | 17.8 | 1 |  | 32.5 |  |  |  |  |
|  | Saltwort-sorghum mixture | Mixture | 17.8 | 1 |  | 22.3 |  |  |  |  |
|  | Sesbania | Monocrop | 11 | 1 |  | 32.2 |  |  |  | Qadir et al., 1996 |
|  | Kallar grass | Monocrop | 11 | 1 |  | 24.6 |  |  |  |  |
|  | Millet rice | Monocrop | 11 | 1 |  | 22.6 |  |  |  |  |
|  | Finger millet | Monocrop | 11 | 1 |  | 5.4 |  |  |  |  |
| Gor-Jordan | Barley | Monocrop | 41 | 1 |  | 1.9 |  |  | 60 | Ammari et al., 2013 |
|  | Barley | Monocrop | 41 | 1 |  | 5.1 |  |  | 55 |  |
|  | Barley | Monocrop | 41 | 1 |  | 2.3 |  |  | 62 |  |
|  | Barley | Monocrop | 41 | 1 |  | 5.2 |  |  | 54 |  |
|  | Finger millet | Monocrop | 41 | 1 |  | 8.2 |  |  | 5.6 |  |
|  | Amaranthus | Monocrop | 41 | 1 |  | 5 |  |  | 0.6 |  |
|  | Sudan grass | Monocrop | 41 | 1 |  | 5 |  |  | 0.4 |  |
|  | Alfalfa (Gor) | Monocrop | 41 | 1 |  | 11.3 |  |  | 2.3 |  |
|  | Kallar grass | Monocrop | 41 | 1 |  | 10 |  |  |  |  |
|  | Salt bush | Monocrop | 41 | 1 |  | 2 |  |  |  |  |
| Texel- Netherlands | Sweet yellow clover | Monocrop | 20 | 1 |  |  |  |  |  | Bruning et al, 2015 |
|  | Alfalfa | Monocrop | 20 | 1 |  |  |  |  |  |  |
| Hafizababd-Pakistan | Oat | Monocrop | 4.3 | 1 |  | 14.9 |  |  |  | Nawaz, 2017 |
| Kesem- Ethiopia | Blue panicum | Monocrop | 13.9 | 2 | 0.08 | 7.6 |  | 3.8 |  | Gelaye et al., 2019 |
|  | Alfalfa | Monocrop | 5.5 | 2 | 0.05 | 6 |  | 1.4 |  |  |
|  | Sudan grass | Monocrop | 13.9 | 2 |  | 6 |  | 1.3 |  |  |
|  | Rhodes grass | Monocrop | 5.5 | 2 | 0.04 | 5.8 |  | 1.3 |  |  |
| Karatas-Turkey | Salicornia | Monocrop | 4.6 | 1 |  | 4.8 | 7.4 |  |  | Yucel et al., 2017 |
|  | Salicornia | Monocrop | 13.3 | 1 |  | 2.9 | 7 |  |  |  |
| Qaroun - Egypt | Kallar grass | Monocrop | 15.4 | 1 | 0.41 |  | 11.2 | 5.24 |  | Tawfik et al., 2013 |
|  | Salt grass | Monocrop | 15.4 | 1 | 0.51 |  | 11.5 | 4.11 |  |  |
|  | Saltmeadow cordgrass | Monocrop | 15.4 | 1 | 0.29 |  | 11 | 2.71 |  |  |
| Werer- Ethiopia | Buffel grass | Monocrop | 16.1 | 1 |  | 37 |  |  |  | Worku et al., 2019 |
|  | Blue panicum | Monocrop | 12.1 | 1 |  | 30 |  |  |  |  |
|  | Sudan grass | Monocrop | 9.8 | 1 |  | 27 |  |  |  |  |
|  | Rhodes grass | Monocrop | 18.1 | 1 |  | 36 |  |  |  |  |
| Faisalabad-Pakistan | Kallar grass | Monocrop | 22 | 5 | 2.39 |  |  | 20 |  | Akhter et al., 2003 |

† EC = Electrical Conductivity

‡ OC = Organic Carbon

§ CP = Crude Protein
